# Three cases of brain metastasis from castration‐resistant prostate cancer

**DOI:** 10.1002/ccr3.2587

**Published:** 2019-12-04

**Authors:** Yohei Shida, Tomoaki Hakariya, Yasuyoshi Miyata, Hideki Sakai

**Affiliations:** ^1^ Department of Urology Nagasaki University Graduate School of Biomedical Sciences Nagasaki Japan

**Keywords:** brain metastasis, castration‐resistant prostate cancer, whole‐brain radiotherapy

## Abstract

Brain metastasis from prostate cancer may be becoming more common and may be associated with occurrence of diffuse systemic metastases.

## Graphical abstract

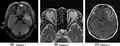

## 1 INTRODUCTION

Brain metastasis associated with prostate cancer is an uncommon phenomenon. Because of its rareness, brain metastasis is usually not considered for follow‐up in prostate cancer. However, improved overall survival in patients with prostate cancer might change this notion. In this report, three cases of brain metastasis from castration‐resistant prostate cancer (CRPC) are described. Whole‐brain radiotherapy (WBRT) was administered in all three cases, and the time from the diagnosis of CRPC to the detection of a brain metastasis was 6, 24, and 29 months, respectively. Overall survival after diagnosis of a brain metastasis was 1, 3, and 5 months, respectively. Brain metastasis in prostate cancer is a rare phenomenon, but it is more frequent in metastatic CRPC patients than in the past.

## 2 CASE HISTORIES

### 2.1 Patient 1

**Figure 1:**
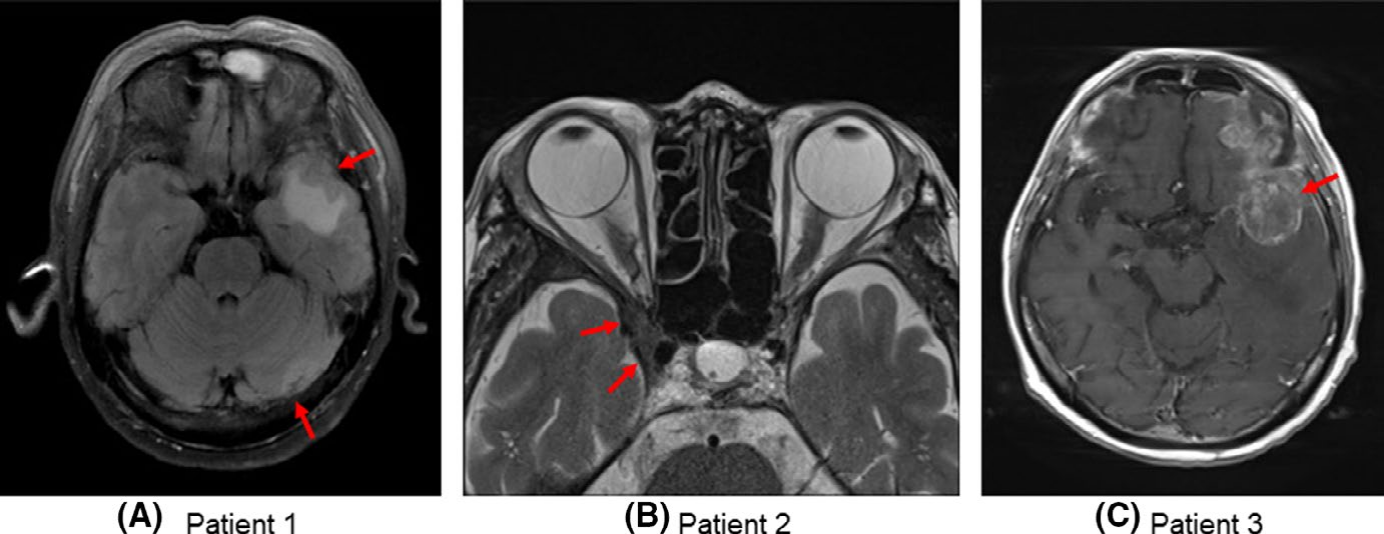
Cranial MRI imaging of three patients. Arrows show metastatic sites. A, Patient 1: Plain cranial MRI showed left middle cranial fossa metastasis. B, Patient 2: Plain cranial MRI showed right cavernous sinus metastasis. C, Patient 3: Gadolinium (Gd) contrast‐enhanced cranial MRI showed a brain metastasis of 3.5 cm in diameter in the left middle cranial fossa

A 64‐year‐old man presented with a prostate‐specific antigen (PSA) level of 25.78 ng/mL. After diagnosis of metastatic prostate cancer (cT4N1M1b) with Gleason score 7 (4 + 3), he was started on combined androgen blockade (CAB) with degarelix and bicalutamide in January 2018. For local control, he underwent intensity‐modulated radiation therapy (IMRT) for the prostate (66 Gy/33 fractions). Although the patient initially responded well to treatment (PSA nadir of 0.039 ng/mL), he was diagnosed with CRPC when we found that his PSA levels had increased 9 months after starting CAB. Abiraterone and prednisolone were administered and resulted in a 50% reduction of PSA. However, despite the reduction of PSA, the disease progressed. He had frequent repeated episodes of severe anemia, and bone marrow aspiration showed a bone marrow metastasis. In April 2019, the patient presented with a severe headache and nausea. Magnetic resonance imaging (MRI) showed multiple brain metastases with major lesions in the left middle cranial fossa (Figure 1A). We administered mannitol, furosemide, and dexamethasone to reduce intracranial pressure and subsequently started WBRT. However, the patient wanted to stop WBRT (the total radiation dose was 9 Gy/3 fractions) and died 1 month after diagnosis of his brain metastasis.

### 2.2 Patient 2

A 53‐year‐old man presented with a PSA level of 36.9 ng/mL. After diagnosis of advanced prostate cancer (cT3aN1M0) with Gleason score 7 (4 + 3), he started on CAB with leuprorelin and bicalutamide in December 2000. CAB was effective for 153 months. Subsequently, his anti‐androgen withdrawal response continued for 11 months, and flutamide was effective for 19 months. However, PSA levels gradually increased, and bone scintigraphy established findings of a bone metastasis. The patient was diagnosed with M1CRPC and was consequently treated with sequential abiraterone, enzalutamide, docetaxel, and cabazitaxel. After three cycles of cabazitaxel therapy, the patient presented at our hospital with a headache and double vision. MRI showed multiple brain metastases with major lesions in the right cavernous sinus (Figure 1B). The patient underwent WBRT (30 Gy/10 fractions) and a fourth cycle of cabazitaxel. After the fourth cycle of cabazitaxel, the patient's Eastern Cooperative Oncology Group Performance Status score decreased to 4, so we ceased cabazitaxel therapy. The patient was transferred to a hospice and died 5 months after diagnosis of his cavernous sinus metastasis.

### 2.3 Patient 3

A 59‐year‐old man presented with a PSA elevation of 7.45 ng/mL. After diagnosis of localized prostate cancer (cT1cN0M0) with Gleason score 7 (3 + 4), he underwent brachytherapy (144 Gy) in April 2007. Two years after brachytherapy, androgen deprivation with leuprorelin was administered because of the recurrence of prostate cancer. However, PSA levels gradually increased, and bone scintigraphy established findings of a bone metastasis. The patient was diagnosed with M1CRPC and was consequently treated with sequential bicalutamide, alpha radium, enzalutamide, abiraterone, docetaxel, and cabazitaxel. After seven cycles of cabazitaxel, the patient's Eastern Cooperative Oncology Group Performance Status score decreased to 3, so we ceased cabazitaxel therapy and administrated dexamethasone. Four months after discontinuation of cabazitaxel, the patient presented at our hospital with a headache, nausea, and aphasia. MRI showed multiple brain metastases with major lesions in the left middle cranial fossa (Figure 1C). We administered mannitol, furosemide, and dexamethasone to reduce intracranial pressure and subsequently started WBRT (30 Gy/10 fractions). Taking into consideration the wishes of the patient and his family, he was transferred to a hospice and died 3 months after diagnosis of his brain metastasis.

## 3 DISCUSSION

Brain metastasis of prostate cancer is a relatively rare phenomenon and remains poorly understood. According to previous reports, the incidence of brain metastasis in prostate cancer is about 3% at most.^1^, ^2^, ^3^, ^4^ However, patients with a non‐adenocarcinoma phenotype who have widely disseminated bone and soft tissue disease are more likely to develop brain metastasis.^5^, ^6^ Hatzoglou et al^4^ reported that the incidence of concurrent bone, lymph node, and liver and/or lung metastases at the time of brain metastasis detection was 95%, 86%, and 76%, respectively. In the present cases, all patients had a concurrent metastasis (ie, bone, lymph node, lung, and/or a liver metastasis) at the time of detection of their brain metastasis (Table 1).

**Table 1:** Patient characteristics

	Patient 1	Patient 2	Patient 3
Age at initial diagnosis (y)	64	53	59
Initial PSA (ng/mL)	25.78	36.9	7.45
Gleason score	7 (4 + 3)	7 (4 + 3)	7 (4 + 3)
Primary treatment	Hormonal therapy	Hormonal therapy	Brachytherapy
CAB^a^ time (mo)	9	153	72
Docetaxel cycles	–	8	4
Cabazitaxel cycles	–	4	7
Age at brain metastasis (y)	66	72	71
Brain metastasis: solitary or multiple	Multiple	Multiple	Multiple
PSA at brain metastasis (ng/mL)	6.94	21.6	291
Concurrent metastases	Bone	Bone, lung, liver, lymph nodes	Bone, lung
Symptoms elicited by brain metastasis	Headache Nausea	Headache Double vision	Headache Nausea Aphasia
Time from the diagnosis of CRPC to the detection of brain metastasis	6	29	24
Overall survival after the diagnosis of brain metastasis	1	5	3

Abbreviations: CAB, combined androgen blockade; LHRH, luteinizing hormone‐releasing hormone; PSA, prostate‐specific antigen.

a CAB was performed with an LHRH analog and bicalutamide.

The most common route by which brain parenchymal metastasis occurs is hematogenous spread. The most common primary tumors that are associated with intraparenchymal brain metastases are lung, breast, melanoma, colon, pancreas, renal, testes, ovary, and cervix.^7^ Intracranial dural metastases usually occur as a direct extension from skull metastases; this explains the high association with breast and prostate cancer, which metastasize commonly to bone. Intracranial dural metastasis can also occur either by direct extension from an epidural metastasis or by hematogenous spread.^8^

In the present cases, the interval from the time of diagnosis of prostate cancer to the detection of a brain metastasis was 15, 146, and 216 months, respectively. Two cases were initially diagnosed as M0 prostate cancer, and the interval until progression to CRPC was substantial. The interval from the time of diagnosis of CRPC to the detection of a brain metastasis was 6, 24, and 29 months, respectively, and overall survival after the diagnosis of a brain metastasis was 1, 3, and 5 months, respectively. Hatzoglou et al^4^ reported that the median overall survival after detection of a brain metastasis in 21 patients was 2.8 months. Overall survival of our patients was comparable with that.

Brain metastasis is more frequent in CRPC patients than in the past because the new approved drugs result in longer survival of metastatic patients.^9^ Actually, we experienced three cases of brain metastasis from CRPC in just half a year. Two of these were detected during cabazitaxel therapy, and the other was detected during abiraterone therapy. Cabazitaxel has been shown to pass the blood‐brain barrier (BBB). Cisternino et al observed a nonlinear accumulation of cabazitaxel in the brains of rats caused by saturation of P‐glycoprotein at the BBB.^10^ However, the efficacy of cabazitaxel for brain metastasis is still unclear because of the rarity of brain metastasis in prostate cancer. Rescigno et al^11^ reported on three cases treated with cabazitaxel for brain metastasis in prostate cancer. Brain metastases were detected before starting cabazitaxel. WBRT at a dose of 30 Gy was administered in all three cases. Two of these showed partial remission with the brain metastasis reduced by half (PSA reduction rate: 60%), and the other showed complete remission with the brain metastasis being undetectable using MRI (PSA reduction rate: 90%). They demonstrated the efficacy of cabazitaxel in treating brain metastases and the tolerability in combination with WBRT. Their report is remarkable in light of a previous retrospective study of 103 patients with brain metastasis that showed that radiotherapy alone was an effective treatment with a median survival of only 3.5 months.^6^ Patients with brain metastasis of CRPC may benefit from cabazitaxel if the brain metastasis is detected prior to cabazitaxel therapy.

Generally, when surgical resection of multiple metastatic brain tumors is considered, the best evidence to date for using this approach is based on traditional principles. Therefore, in patients with good functional status and multiple lesions, resection of large, dominant, symptomatic metastases (up to 2‐3) may benefit performance status without worsening survival.^12^ Patients with a solitary resectable symptomatic intracranial dural metastasis, controlled systemic cancer, and acceptable surgical risk should be considered for resection as first‐line therapy.^8^

In the present cases, surgical debulking was considered prior to WBRT but abandoned because of the high number (>5) of metastases and the uncontrolled systemic cancer. WBRT is still preferred in the setting of numerous brain metastases, carcinomatous meningitis, or primary histologies prone to micrometastatic disease. In these instances, stereotactic radiosurgery (SRS) often cannot effectively target all the disease.^12^ For these reasons, the present cases were treated with WBRT alone. In our present cases, the symptoms elicited by the patients' brain metastases were alleviated using WBRT. However, the prognosis of the present cases was quite bleak. In all CRPC patients with concurrent widely disseminated bone and soft tissue disease, the possibility of a brain metastasis should be considered. A breakthrough in the treatment of brain metastasis of CRPC is needed.

## CONFLICT OF INTEREST

The authors declare that they have no competing interests.

## AUTHOR CONTRIBUTIONS

YS: YS contributed to the conception, design, and drafting of the manuscript. YS wrote the manuscript. TH: TH contributed to the critical revision of the manuscript. YM: YM contributed to the writing and critical revision of the manuscript. HS: HS contributed to the design and critical revision of the manuscript. All authors read and approved the final manuscript.

## INFORMED CONSENT

Written informed consent was obtained from the patients.
